# Laser-Induced Graphene for Heartbeat Monitoring with HeartPy Analysis

**DOI:** 10.3390/s22176326

**Published:** 2022-08-23

**Authors:** Teodora Vićentić, Milena Rašljić Rafajilović, Stefan D. Ilić, Bojana Koteska, Ana Madevska Bogdanova, Igor A. Pašti, Fedor Lehocki, Marko Spasenović

**Affiliations:** 1 Center for Microelectronic Technologies, Institute of Chemistry, Technology and Metallurgy, National Institute of the Republic of Serbia, University of Belgrade, 11000 Belgrade, Serbia; 2 Faculty of Computer Science and Engineering (FCSE), “Ss. Cyril and Methodius” University, 1000 Skopje, North Macedonia; 3 Faculty of Physical Chemistry, University of Belgrade, 11158 Belgrade, Serbia; 4 Faculty of Informatics and Information Technologies, Slovak University of Technology, 842 16 Bratislava, Slovakia; 5 Institute of Measurement Science of the Slovak Academy of Sciences, 841 04 Bratislava, Slovakia

**Keywords:** bioinformatics, biomedical electronics, biomedical materials, biomedical signal processing, biosensors, medical information systems, programming, software performance, wearable sensors

## Abstract

The HeartPy Python toolkit for analysis of noisy signals from heart rate measurements is an excellent tool to use in conjunction with novel wearable sensors. Nevertheless, most of the work to date has focused on applying the toolkit to data measured with commercially available sensors. We demonstrate the application of the HeartPy functions to data obtained with a novel graphene-based heartbeat sensor. We produce the sensor by laser-inducing graphene on a flexible polyimide substrate. Both graphene on the polyimide substrate and graphene transferred onto a PDMS substrate show piezoresistive behavior that can be utilized to measure human heartbeat by registering median cubital vein motion during blood pumping. We process electrical resistance data from the graphene sensor using HeartPy and demonstrate extraction of several heartbeat parameters, in agreement with measurements taken with independent reference sensors. We compare the quality of the heartbeat signal from graphene on different substrates, demonstrating that in all cases the device yields results consistent with reference sensors. Our work is a first demonstration of successful application of HeartPy to analysis of data from a sensor in development.

## 1. Introduction

Wearable sensors are an expanding field of research, with growing applications in telehealth [1], fitness tracking [2], and mass casualty incident management [3]. Various shapes of wearable sensors of physiological parameters are under development, such as patches [4,5,6,7], bands [8,9,10], or watches [11]. Physiological parameters being monitored include heart rate, blood pressure, electrocardiogram (ECG), sweat composition, and breathing rate and volume. Aside from making the hardware of a novel sensor, engineers need to apply a software component to analyze the collected data and extract the value of the target physiological parameter.

Heart rate signals from photoplethysmogram (PPG) measurements are known to contain noise, large amplitude variations and a broad variation of peak morphology [12]. The HeartPy toolkit for Python is an excellent open source package that was designed mainly for evaluating heart rate signals from PPG data [13]. The toolkit has been applied to analyze heart rate data from commercially available devices [14,15,16] and even smartphone cameras [17]. Nevertheless, to the best of our knowledge, although HeartPy is an ideal tool for developers of novel heart rate sensors, the toolkit has not been used in conjunction with sensors that are under development. In this paper, we describe the application of HeartPy to analysis of heart rate data gathered with a novel graphene-based sensor.

The discovery of graphene [18] has spurred an impressive number of research papers, due to the material’s unique and favorable electronic, optical, chemical and mechanical properties. Graphene is abundant, thin, flexible, electrically conductive, and, subjective to tailoring, its chemical reactivity by geometry and functionalization. Graphene has been used to produce various types of environmental and physiological sensors [19,20,21,22]. Among the numerous types of graphene, classified by the method by which they are made [23], laser-induced graphene (LIG) has most recently emerged as a platform for sensors that can be made in custom shapes and dimensions with a facile fabrication process [24]. Early proof-of-concept papers have shown that LIG can be used to measure human heartbeat [25,26,27]. To measure heartbeat, the piezoresistive nature of LIG is utilized, with a device attached to a position on the body where vein pulsing can be detected. The purpose of our study is to demonstrate the feasibility of applying HeartPy to novel heartbeat sensors in development. We demonstrate that LIG heartbeat sensors can be used in conjunction with HeartPy to obtain heart rate parameters that are in agreement with those measured with a reference sensor or estimated manually. Furthermore, we show that the sensors work with as-made LIG on polyimide (PI), but also on LIG on PI protected with a top layer of polydimethylsiloxane (PDMS), as well as LIG completely transferred from PI to PDMS. These observations are important for the practical use of LIG devices in wearable physiological parameter monitoring.

Heartbeat signals contain the value of numerous physiological parameters that are important health indicators. The most commonly extracted value, heart rate, is defined as the number of heartbeats per minute. Heart rate variability (HRV) is the fluctuation in the time intervals between adjacent heartbeats [28]. Another relevant parameter is the interbeat interval (IBI), that provides information on the spacing between heart beats. Interestingly, heartbeat signals also contain information on the person’s breathing rate (BR). To date, BR from heartbeat has only been measured with optical methods [29,30]. Our work shows that sensors operating on the principle of piezoresistivity can also be used to extract BR from heartbeat. Our work is the first demonstration of the HeartPy toolkit operation on a custom-built heartbeat sensor, which paves the way to practical use of this package, as an enabling tool for sensor developers.

The paper is organized as follows. In Section 2 we describe the production of LIG and material characterization. We also describe the measurements of heartbeat and HeartPy signal analysis. In Section 3 we show the results of the material characterization, the heartbeat measurements, and HeartPy analysis. In Section 4 we discuss the results, especially in the context of practical use of the combination of HeartPy with graphene sensors.

## 2. Experimental

**Figure 1:**
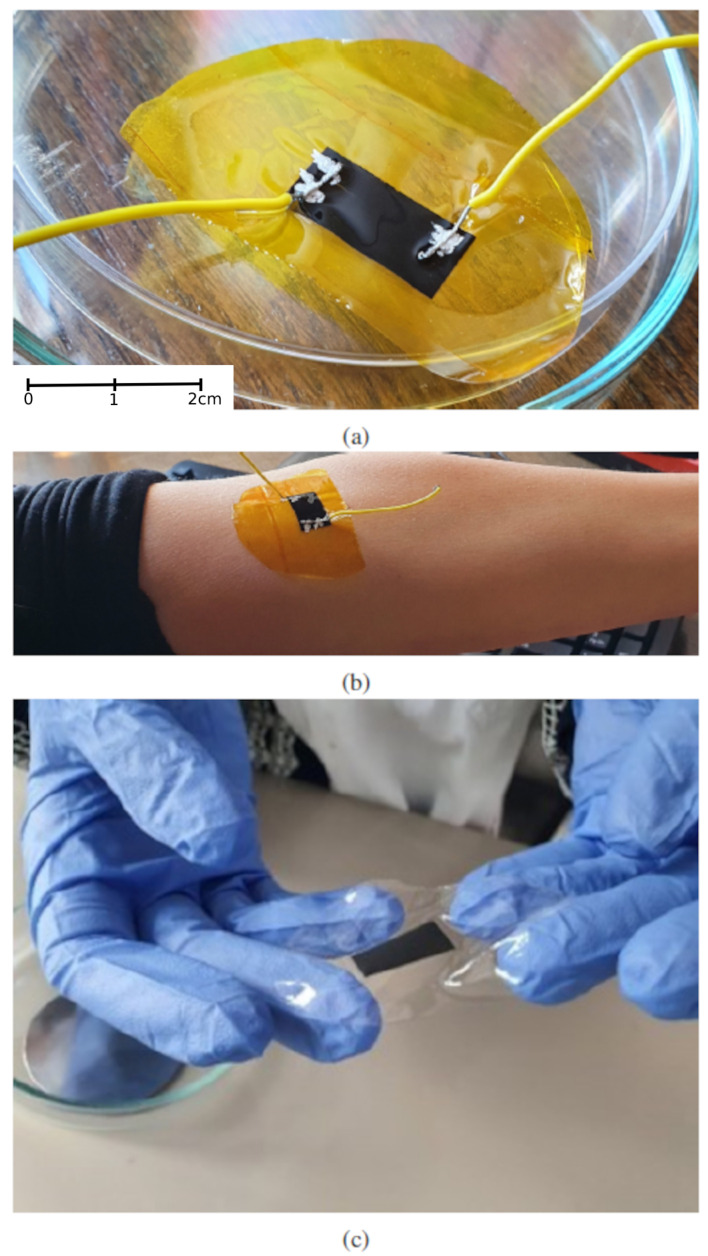
Photographs of the graphene sensors. (**a**) Graphene on a PI substrate, covered with PDMS seen as a glossy top layer, and contacted with two wires at sensor ends. The wires were attached to the graphene with silver paste. (**b**) The sensor with wires on a subject’s forearm, at the position of the median cubital vein. (**c**) Graphene completely transferred to PDMS, with the PI substrate removed.

LIG was produced by scanning a $CO_{2}$ laser beam across the surface of polyimide tape, as in [31]. The laser used was a DBK FL-350 with maximum power of 60 W, with power set to 20%, scanning speed 900 mm/s, and resolution of 600 DPI. The devices were formed by laser-writing LIG in the shape of rectangles with dimensions 1 × 2 cm. Electrical contacts were made to the LIG by attaching wires with silver paste at the ends of the device, as depicted in Figure 1a. For devices with PDMS, we used the Sylgard 184 two-component elastomer kit (10:1 mix ratio). After mixing, PDMS was applied on top of the graphene with spin coating, at a rotation of 1000 rpm. The thickness of PDMS at that rotation speed is 50 μm. After curing for 1 h at a temperature of 60 degrees, the LIG device is either used on the PI substrate with the PDMS protection layer, or the PI tape is removed to leave graphene embedded in PDMS, as depicted in Figure 1c.

Scanning electron microscopy (SEM) with energy dispersive X-ray spectroscopy (EDS) was done with a PhenomProX electron microscope (Phenom, Thermo Fisher Scientific, Waltham, MA, USA). Reported elemental compositions are obtained as the average over several positions recorded with a magnification of ×1000, where EDS was performed over the entire field of view. The Raman spectra of the samples were recorded with a DXR Raman microscope (Thermo Fisher Scientific, Waltham, MA, USA). The samples were excited with a diode laser at a wavelength of 532 nm and a power of 10 mW focused on a 2.1 μm spot on the surface. Spectra were obtained as averages of three measurements from different positions on each sample (10 exposures, 10 s each per position). Recorded Raman spectra were automatically corrected for fluorescence.

Graphene devices were attached by adhesion to the arm of a still subject at the position of the median cubital vein, as in Figure 1b. We have found that the median cubital vein is the optimal position for placing the graphene sensor. We have also placed the sensor on the wrist and on the chest, where blood pumping can be registered as motion of the surface of the skin, however local body geometry at the position of the median cubital vein proved to be optimal for reliable sensor adhesion. The wires were connected to a Keithley 2450 SMU, which was interfaced to a desktop computer. Measurements were performed in the constant current mode with current set to 1 mA, and the voltage was measured over a period of several minutes. As a reference, the heart rate was measured with a free app installed on a smartphone and recorded in parallel. The breathing rate was manually estimated by counting breaths during the measurement interval.

The signals were analyzed with the HeartPy Python Toolkit [13]. Two different API were used to preprocess the LIG signal and calculate the needed parameters—heartpy and heartpy.filtering. The main used function is process(), contained in the heartpy module. We also used the plotter() function in order to obtain the visualisation of the signal and the accepted and rejected peaks. The preprocessing part is set by the heartpy.filtering module, i.e., the function heartpy.filtering.filter-signal().

The obtained signals differ in quality from one measurement instance to another, depending on two factors—on the exact sensor positioning on the subject’s arm, and on the stillness of the subject. In the case of an extremely noisy signal, preprocessing was performed before applying the HeartPy process() function. Noisy input signals were normalized to zero mean and unit variance. Filtering techniques such as low pass filtering and high pass filtering were applied to eliminate high frequency noise from the signal. Specifically, 8th order Butterworth band-pass filtering was performed [32].

In order to identify peaks in the heartbeat with HeartPy, the process() function starts by calculating the moving average using a window of 0.75 s on both sides of each data point. Next, regions of interest (ROI) are defined as regions between the two points of intersection where the amplitude of the signal is larger than the moving average. The process() function uses two approaches to identify the location of a peak. In the first approach, the maximum value in the marked ROI is taken as the peak position, while in the second approach a univariate spline is used to upsample and interpolate the ROI, and solved for its maximum [12]. A special case occurs with signal clipping, which may occur in cases of extreme noise. In the case of clipping, the algorithm detects the onset and end of clipping segments and uses spline interpolation to make a reconstruction of the waveform. The best solution is found by minimizing the standard deviation of peak–peak intervals. The heart rate (beats per minute—BPM) is computed and evaluated together with the standard deviation of peak–peak intervals. The BPM value lies within a predefined range which can be customized by the user (by default BPM is set between 40 and 180) [33].

## 3. Results

**Figure 2:**
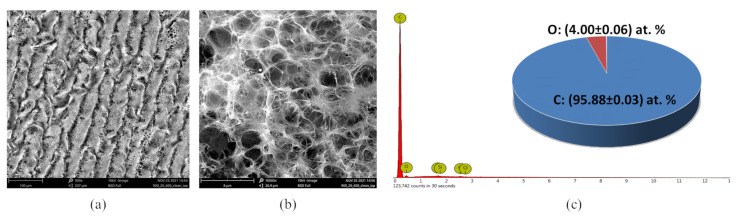
SEM and EDX characterization of LIG on PI. (**a**) SEM at a magnification of ×500, where lines along which the laser passed can be observed. (**b**) SEM at a magnification of ×10,000, where a highly developed porous network is seen. (**c**) EDS spectra and the C and O content in LIG. Trace elements such as Si and Cl were left out of the pie chart, because those are elements found in the substrate and not in the graphene sample itself.

SEM imaging of LIG on PI reveals a highly developed porous network (Figure 2a,b). EDS reveals that the interconnected network is predominantly composed of C and O (Figure 2c), with uniform elemental distribution across the network. Oxygen concentration is low, which indicates weak partial oxidation of LIG. Besides C and O, traces of Cl, (0.06 ± 0.02) at %, and Si, (0.06 ± 0.01) at %, are present in the sample.

**Figure 3:**
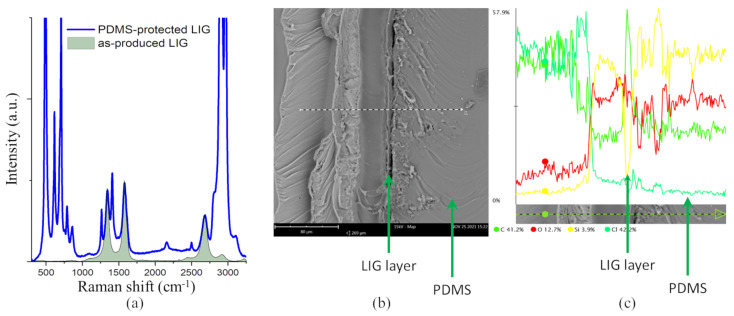
Raman spectra, SEM, and EDX characterization of PDMS-covered LIG layer. (**a**) Raman spectra of the as-produced LIG sample before (shaded) and after covering with PDMS protective layer. (**b**) SEM image of a cross-section of the PDMS-covered LIG layer on PI. (**c**) EDS line scan across the dashed white line depicted in panel (**b**). Different colored lines indicate different elements, as stated in the legend below the panel.

Raman spectroscopy of LIG shows typical features of graphene, with clearly visible D and G bands in the region 1000–1750 cm^−1^, and a very well developed 2D region (2250–3000 cm^−1^, Figure 3a. Once covered with PDMS, the spectral features of LIG are still visible, but overlapped with the spectrum of PDMS, which is highly intense. There are no distinct changes of the relative intensities of D and G bands of LIG upon covering with PDMS, which suggests that the transfer to PDMS was performed without adding further defects to the graphene. A SEM image of a mechanically made cross-section of a PDMS-covered LIG layer suggests good adhesion with no obvious delamination (Figure 3b), which can be a concern at the contact between the LIG layer and the underlying PI substrate. EDS scans along lines across the material interfaces, such as along the white-dashed line depicted in Figure 3b, clearly reveal the LIG layer as a sharp peak in the carbon concentration profile, Figure 3c. The thickness of the layer is around 8 μm. The PDMS and PI layers contain substantially less C than the graphene layer, but contain more Si.

HeartPy analysis returns signal visualization in the form of a graph containing peaks marked with circles, either green or red. Green circles mark peaks that are selected for the heart rate analysis, while red circles mark peaks that the algorithm finds to be erroneous. In order to detect the aforementioned peaks, this function uses an adaptive threshold to accommodate for morphology and amplitude variation in the signal waveform, followed by an outlier detection and rejection.

The heartpy module process() function returns several parameters, including HR and BR. The HR (BPM) is an aggregate measure, calculated as the average beat–beat interval across the entire analyzed signal (segment), resulting in a very robust algorithm. The BR by HeartPy is obtained by exploring the property of the heart that the frequency of the heartbeat is strongly influenced by breathing. There is a strong relationship between the breathing and the intervals between the heartbeats (R–R intervals). HeartPy upsamples the list of detected R–R intervals by interpolation, followed by extraction of breathing peaks in the signal [12].

The HeartPy process() function also returns 11 more parameters—7 are in the time domain, and 4 are non-linear measurements. The time-domain parameters are computed on the detected and accepted peaks in the R–R intervals. These time-domain indices of HRV quantify the amount of variability in measurements of the inter-beat interval (IBI), which is the time period between successive heartbeats. SDNN is the standard deviation of IBI, measured over normal sinus beats (abnormal beats, like ectopic beats have been removed), while SDSD is the related standard deviation of successive R–R interval differences, only representing short-term variability. RMSSD is the root mean square of successive differences between normal heartbeats. It is obtained by calculating and squaring each successive time difference between heartbeats and averaging the result before the square root of the total is produced. Parameters pNN50/pNN20 represent the percentage of adjacent normal sinus beats that differ from each other by more than 50/20 ms [34]. The function process() produces another statistical metrics—the median absolute deviation of R–R intervals (MAD). We used this metrics to compare the data values in the all three experiments—the larger the MAD, the greater the distance between the normal heartbeats. The other 4 parameters are the non-linear measures that quantify the unpredictability of a time series, which results from the complexity of the mechanisms that regulate HRV. Poincare plot analysis is represented with a scatter plot by plotting every R–R interval against the prior interval. The S, SD1, SD2, and SD1/SD2 parameters are extracted from a Poincare plot, that can be analysed by fitting an ellipse to the plotted points. After fitting the ellipse, three non-linear measurements, S, SD1, and SD2 can be derived. The area of the ellipse which represents total HRV is represented by the parameter S. The standard deviation of the distance of each point from the y = x axis (SD1), specifies the ellipse’s width. SD1 measures short-term HRV in ms. The standard deviation of each point from the y = x + average R–R interval (SD2) specifies the ellipse’s length. SD2 measures short- and long-term HRV [34].

In a nutshell, the used HeartPy process() function produces the following parameters:

- BPM—the number of heartbeats per minute, the HR;
- Breathing rate in Hz, that multiplied by 60 gives the number of breaths per minute, the BR.

*Time-domain measures*

- IBI—inter-beat interval, the mean distance of intervals between heartbeats;
- SDNN—the standard deviation of intervals between heartbeats (R–R intervals);
- SDSD—the standard deviation of successive differences between adjacent R–R intervals;
- RMSSD—the root mean square of successive differences between adjacent R–R intervals;
- PNN50/PNN20—the proportion of differences greater than 50 ms/20 ms;
- MAD—median absolute deviation of R–R intervals;

*Non-linear measures*

- SD1, SD2, S, SD1/SD2—Poincare plot analysis

The experiments were conducted with a graphene sensor attached to the forearm of a still sitting subject. Reference values were measured with a smartphone app that uses the device camera to record HR, applying optical methods. In this study experiments were conducted with 3 different versions of the graphene patch. The presented results are representative examples of signals obtained from the three different patch versions. The results were reproducible in 90% of the cases. The reproducibility and the quality of processed signals mainly depend on the precise positioning and fixture of LIG-based sensors. The tighter the sensor is applied to the cubital vein, the higher the signal quality.

The experiments are classified as follows:

- First experiment—pure laser-induced graphene on PI;
- Second experiment—graphene on PI protected by a PDMS layer on top;
- Third experiment—graphene completely transferred to PDMS.

**Figure 4:**
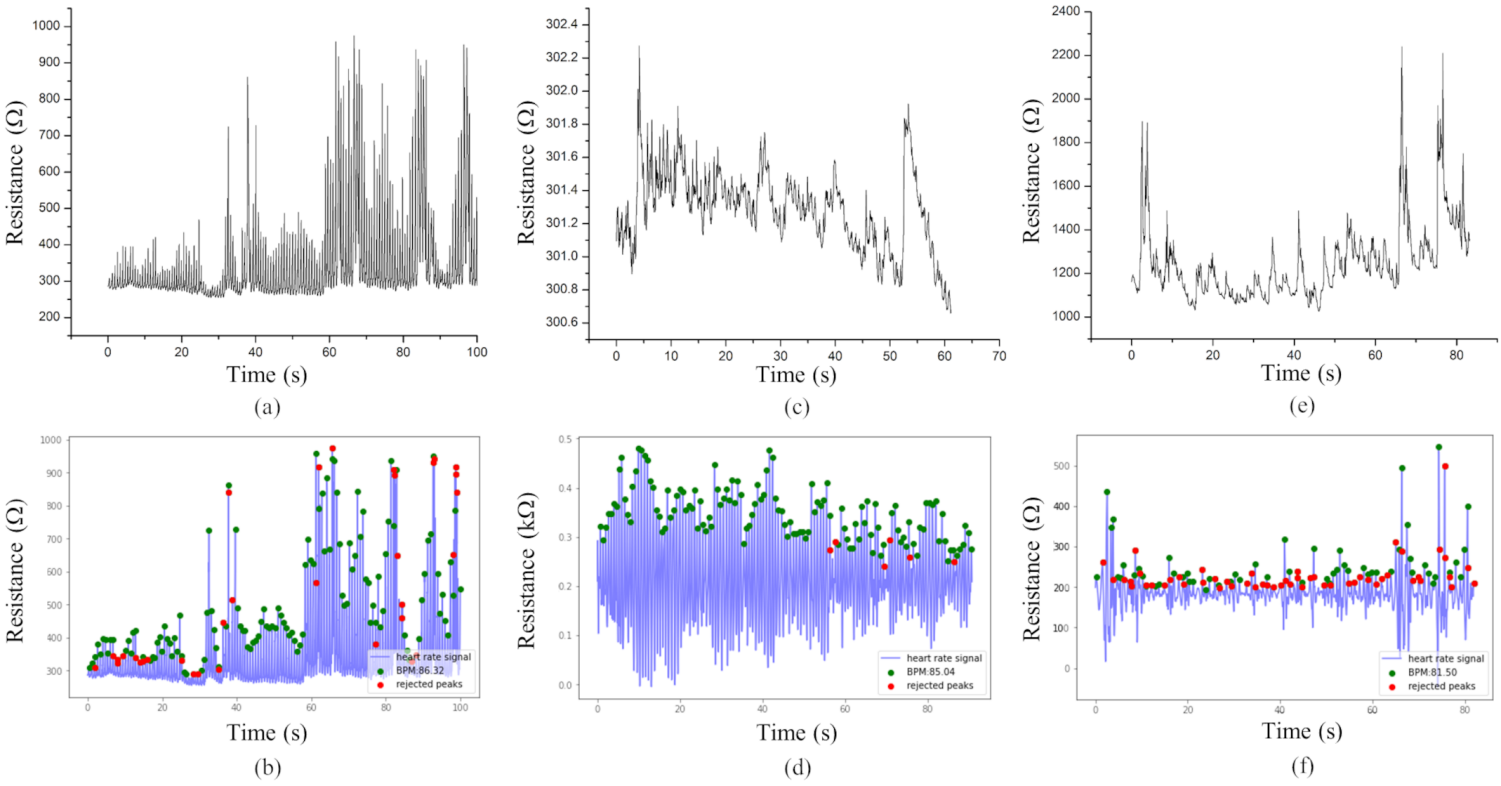
Measurements and analysis of heartbeat. (**a**) Resistance variation in time, as vein pulsing is measured with LIG on polyimide. (**b**) The same data as in (**a**), after processing with HeartPy. Green points mark peaks that were selected for heart rate analysis, while red points mark peaks that the algorithm found to be erroneous. (**c**) Resistance variation in time, as vein pulsing is measured with LIG on polyimide, protected with a PDMS layer on top. (**d**) The same data as in (**c**), after processing with HeartPy. (**e**) Resistance variation in time, as vein pulsing is measured with graphene completely transferred to PDMS. (**f**) The same data as in (**e**), after processing with HeartPy.

The measurements from the first experiment are depicted in Figure 4a. A periodic variation in resistance is observed as the vein pulses. Applying the HeartPy toolkit analysis method to the data yields the graph presented in Figure 4b.

The measurements and the analysis for the second experiment are presented in Figure 4c,d, and the third experiment in Figure 4e,f.

Table 1 presents the difference between the HR obtained by the HeartPy toolkit and the measured HR by the smartphone commercial application, the BR produced by HeartPy and the real BR and the number of rejected peaks in the all three experiments. It is notable that the HeartPy analysis proves to be robust in the case of HR and BR, working effectively across all three experiments, regardless of the noise or signal drift present in the original data. These results show that LIG-based HR sensors can be used in emergency situations with patch-like devices that continuously monitor and analyze health status of an injured person. The BR measured with LIG in conjunction with HeartPy is usable in triage, since the measured values differ from reference values by no more than 2 breaths per minute, which does not interfere with the START triage procedure [35]. The number of rejected peaks varies from experiment to experiment, which is probably attributable to the stillness of the subject and the exact positioning of the sensor across the vein. Although nearly 50% of the peaks were annotated as erroneous in the third experiment, the obtained values for HR and BR agree with reference measurements, once again pointing to the robustness of the HeartPy algorithm used in conjunction with the aforementioned patch-like device.

An overview of the 13 extracted parameters from HeartPy analysis toolkit, with the corresponding values for the all three experiments are presented in Table 2.

It is evident that the values of the HRV time-domain and non-linear measurements from the process() function mostly have risen as the graphene is further treated. The standard deviations SDNN, SDSD, and the median absolute deviation (MAD), show less reliable data in the second and the third experiment, compared to the first one. Nevertheless, the obtained values for HR and BR are within the acceptable range, as presented in Table 1.

The as-made LIG devices (first experiment) yield signals of the best quality of the three experiments, whereas the LIG devices completely transferred to PDMS (third experiment) yield signals with highest indicators related to noise. Since the Raman spectra measurements have indicated that the quality of the graphene does not decrease with treatment, the origin of the decrease of signal quality in the composite sensor is likely due to imperfect conformity of graphene to the PDMS layer and the increased mechanical stiffness of the samples that include PDMS.

The obtained HRV time-domain measures and the Poincare analysis measurements are within the range expected from heartbeat analysis [34].

## 4. Discussion

In the above sections, we have shown that LIG wearable sensors can be used to reliably collect physiological data that can be processed with an easily accessible HeartPy open source toolkit. Wearables can be used to perform several basic functions: sense, analyze, store, transmit, and utilize data [36]. Processing of the recorded physiological signals can be done either on the sensor (wearer) or at a remote location where higher processing power is available (e.g., healthcare provider). Proprietary personalised responses can be generated and communicated to the individual as a response to a changing health situation. Wearable patch devices can be applied even in mass casualty situations, where there is limited access to an Internet (mobile network) connection, low visibility due to smoke, or high ambient noise. In such conditions, a low-profile graphene-based patch sensor can be used to provide on-board unobtrusive longitudinal sensing and processing of heart rate, breathing rate and possibly SpO2 data. The sensor can be integrated into a START triage procedure [35] to generate appropriate and easily spottable audio and visual response (flashing colours) in case of deterioration of a patient’s or victim’s health status. The final design of a patch-like sensing device should be disposable, responsive, multifunctional active device enabling producing several physiological signals (e.g., HR, BR, SpO2), which is possible with the proposed graphene-based sensors.

On their part, the graphene sensors have shown to be easy to make, inexpensive, and operable on various substrates, including PDMS which is often used as a platform for wearable electronic health patches. LIG heartbeat sensors are very sensitive, and can suffer from noise that is induced when the subject moves. This high-frequency noise needs to be removed in post-processing with functions such as filtering, which introduces extra processing steps.

The main contribution of our work is that it demonstrates that a free, open-source toolkit for heartbeat analysis such as HeartPy can be used in conjunction with any heartbeat sensor, including ones in development, such as the one we developed from graphene. This will allow laymen to delve into developing sensor applications, by using, e.g., commercial off-the-shelf piezoelectric thin film sensors in conjunction with HeartPy. The combination of easy to make, inexpensive graphene heartbeat sensors and a widely available open access HeartPy toolkit for data analysis has potential for use by medical researchers or in finalized devices.

## 5. Conclusions

To conclude, we have demonstrated effective heartbeat sensors that consist of graphene-based sensing elements on several flexible and biocompatible substrates, in conjunction with analysis with the open-source package HeartPy toolkit for Python. The LIG signals were processed with two HeartPy modules—heartpy and heartpy.filtering. The HeartPy package is an excellent tool that was designed mainly for evaluating heart rate signals from PPG data. Nevertheless, we have shown that HeartPy analysis can be conducted on the LIG signals as well, especially in obtaining the HR and BR parameters.

This combination of hardware and software presents a relatively low barrier of entry for novice developers of heartbeat sensors, opening a path for widespread experimentation and application. Future work may address the biocompatibility, reusability, and price of the substrates and materials used, in order to bring solutions closer to the market. In addition, future research may exploit the advantages of big data to optimize the use of HeartPy with graphene-based sensors.

## Figures and Tables

**Table 1:** HR (HeartPy and application), BR (HeartPy and counted) and number of rejected peaks (HeartPy) in all three experiments.

Experiment	HeartPy HR	app HR	HeartPy BR	Real BR	Rejected Peaks
first	86.3	86	10.0	12	33/156
second	85.0	85	14.4	14	6/130
third	81.5	84	12.1	12	54/122

**Table 2:** Values of the extracted parameter with HeartPy for all three experiments.

	First	Second	Third
HR (BPM)	86.3	85.0	81.5
BR	0.167 × 60 = 10.0	0.234 × 60 = 14.4	0.20 × 60 = 12.1
**HRV time-domain measurements**			
IBI	695.1	705.5	736.2
SDNN	52.3	57.9	153.3
SDSD	31.1	51.9	109.9
RMSSD	49.9	75.9	214.04
PNN20	0.7	0.7	0.9
PNN50	0.3	0.4	0.8
MAD	31.9	40.8	122.5
**Non-linear measurements**			
SD1	35.2	53.7	149.4
SD2	62.4	60.7	154.0
S	6895.6	10,245.9	72,295.4
SD1/SD2	0.6	0.9	0.9

## Data Availability

Data available on request from the corresponding author.

## Abbreviations

The following abbreviations are used in this manuscript:

ECG	Electrocardiogram
PPG	Photoplethysmogram
LIG	Laser-induced graphene
PI	Polyimide
PDMS	Polydimethylsiloxane
HRV	Heart rate variability
HR	Heart rate
BR	Breathing rate
SEM	Scanning electron microscopy
EDS	Energy dispersive X-ray spectroscopy
